# Antiseptics as adjuncts to scaling and root planing in the treatment of periodontitis: a systematic literature review

**DOI:** 10.1186/s12903-020-01127-1

**Published:** 2020-05-18

**Authors:** Egle Ramanauskaite, Vita Machiulskiene

**Affiliations:** Clinic of Dental and Oral Diseases, Faculty of Dentistry, Lithuanian University of Health Sciences, Eiveniu 2, 5009 Kaunas, Lithuania

**Keywords:** Periodontitis, Antiseptics, Treatment, Review

## Abstract

**Background:**

Periodontitis is microbially-associated, host-mediated inflammatory condition that results in loss of periodontal attachment. The goals of periodontal therapy include arresting the disease progression, establishing healthy, stable, maintainable periodontal conditions. A fundamental strategy of treating periodontitis is scaling and root planning (SRP), however its efficacy may be restricted in areas inaccessible for mechanical instrumentation. As periodontitis is infectious in nature, it might be helpful to use additional antimicrobial adjuncts, in order to eliminate or inactivate pathogenic microflora. The aim of this study is to evaluate the current evidence regarding the potential clinical benefits of using additional antiseptics for SRP in nonsurgical periodontal therapy.

**Methods:**

An electronic literature search was conducted in the MEDLINE (Ovid) and Cohrane Central Register of Controlled Trials (CENTRAL) databases for articles published between January 1, 2000 and September 22, 2019. Randomized controlled clinical trials in English that compare the effectiveness of one or more antiseptic agents as adjuncts to SRP with a follow-up of ≥6 months were included. A meta-analysis using the random-effects model was performed on the selected qualifying articles.

**Results:**

The search resulted in 12 articles that met the inclusion criteria. Based on the vehicle employed to deliver the antiseptic agent, studies were divided into adjunctive sustained-release antiseptics (gels, chips and varnish) and adjunctive irrigation with antiseptics. The meta-analysis demonstrated significant improvements in probing depth (PD) reduction (*p* = 0.001), clinical attachment level (CAL) gain (*p* = 0.001), and bleeding on probing (BOP) values (*p* = 0.001) following the adjunctive subgingival application of sustained-release antiseptics. Additional subgingival irrigation with antiseptics failed to show significant improvements in PD (*p* = 0.321), CAL (*p* = 0.7568), or BOP values (*p* = 0.3549) over SRP alone.

**Conclusions:**

Adjunctive subgingivally delivered antiseptics with a sustained-release delivery have significant clinical benefits compared to SRP alone.

## Background

Periodontitis is a chronic multifactorial inflammatory disease associated with dysbiotic plaque biofilms. It is clinically characterized by progressive attachment and alveolar bone loss [1]. The number of people affected by periodontitis has grown substantially, increasing the global burden of the disease [2].

The 2017 World Workshop on the Classification of Periodontal and Peri-Implant Diseases and Conditions has brought new updates to the previous internationally accepted periodontal disease classification (Armitage 1999 [3]). According to the new classification, the disease phenotypes previously recognized as “chronic” and “aggressive” were grouped under one category, “periodontitis,” and further characterized based on a multidimensional staging and grading system [1, 4].

Despite the updates, treatment goals remain unchanged: arresting the disease’s progression; preserving healthy, stable, and maintainable periodontal conditions; and, if possible, regenerating lost tissues.

According to the cause-related therapy concept, SRP is as a cornerstone of periodontal therapy [5]. Its primary goal is to remove soft and hardened microbial deposits from the pathologically exposed root surfaces [6]. Ideally, periodontal therapy should also reduce or eliminate the pathogenic species that cause and/or sustain periodontal diseases [6].

However, this therapy is technically demanding and has certain limitations. Bacterial plaque cannot be sufficiently eliminated from deep pockets, intrabony defects, or furcation areas. It also depends on the operators’ manual skills and on various patient-related factors (e.g., patients’ smoking status and systemic diseases). Up to 30% of the total surface area of subgingivally debrided roots may be covered with residual calculus [7]. Therefore, it is important to use adjunctive antimicrobial chemotherapeutic agents to eliminate or inactivate pathogenic microflora in sites where mechanical instrumentation is invidious.

Recent studies show that periodontal therapy outcomes may be enhanced by using additional systemic [8, 9] or local antibiotics [10–12] and antiseptics [13–15].

The emerging global public health issue of bacterial resistance has increased the number of warnings against the unrestricted use of antibiotics to treat periodontal disease [16]. Therefore, systemic antibiotics in periodontitis should be restricted to certain patients under certain periodontal conditions (stages III-IV, grade C, “active” forms, “refractory”, and “recurrent” forms of a disease), and they should be used rationally while following optimal protocols [17].

Local antibiotics suffer from several potential problems, including an insufficient spectrum of antimicrobial activity, risks of producing an antibiotic-resistant microbiota, and high acquisition costs [18].

Antiseptics are chemical agents that can destroy microorganisms on live tissues. Antiseptics have some beneficial properties compared to systemic or local antibiotics [18]. In particular, they have a more extensive activity spectrum. Furthermore, the possibility of resistance formation is reduced by having multiple intracellular targets [18].

The aim of this study is to estimate the current evidence evaluating the potential clinical benefits (in terms of probing depth (PD), bleeding on probing (BOP) reduction and/or clinical attachment level (CAL) gain) of using additional local antiseptics to conventional SRP in nonsurgical periodontal therapy.

## Methods

This systematic analysis report adhered to the Preferred Reporting Items for Systematic Review and Meta-Analyses (PRISMA) statement [19].

### Protocol and registration

The review was registered in PROSPERO, an international prospective registry of systematic reviews, under number CRD42018086904. The analysis methods and inclusion criteria were specified in advance and documented in a protocol and are accessible through the following link:

https://www.crd.york.ac.uk/prospero/display_record.php?RecordID=86904

### Focus question

The following focus question was developed according to the population, intervention, comparison, and outcome (PICO) study design: Does the adjunctive application of antiseptics to SRP have additional clinical benefits compared to SRP alone in treating periodontitis?

Population: Chronic periodontitis patients;

Intervention (test): SRP plus adjunctive antiseptics;

Comparison (control): SRP alone or plus a placebo;

Outcome: The primary outcome variable was the changes in pocket-probing depths (PD); secondary outcome variables included changes in clinical attachment level (CAL) and/or bleeding on probing (BOP).

### Information sources

A systematic electronic literature search was conducted in MEDLINE (Ovid) and Cohrane Central Register of Controlled Trials (CENTRAL) databases. Studies published between January 1, 2000 and September 22, 2019 were searched. An electronic search was supplemented by a manual search of the following journals: *International Journal of Periodontics and Restorative Dentistry, Journal of Clinical Periodontology, Journal of Periodontology*, and *Journal of Periodontal Research*.

Scanning of the bibliographies of all publications included into this review was performed for potentially relevant articles.

### Search

The keywords used to search the selected electronic databases included the following Specific Medical Subject Headings (MeSH) terms: (“periodontitis” [Mesh] OR “periodontal disease” [Mesh] OR “chronic periodontitis” [Mesh] AND (“treatment” [Mesh] OR “therapy” [Mesh] OR “antiseptics” [Mesh] OR “scaling and root planning” [Mesh] OR “subgingival irrigation” [Mesh] OR “non-surgical therapy” Mesh]).

### Selection of studies

The resulting articles were revised by two independent reviewers (E.R. and V.M.), based on the inclusion criteria. Disagreements regarding inclusion during the first and second stages of the study selection were resolved by discussion. The agreement level between the reviewers regarding study inclusion was calculated using unweighted κ statistics.

### Inclusion and exclusion criteria

During the first stage of study selection, the titles and abstracts were screened and evaluated according to the following inclusion criteria:

- Randomized controlled clinical trials (RCTs) comparing the effectiveness of one or more antiseptic agents as adjuncts to SRP;
- An antiseptic was applied to periodontal pockets only at the time of SRP;
- A control group received the same SRP as the test group either alone or with a placebo;
- SRP was carried out with both ultrasonic and hand instruments;
- A follow-up no less than 6 months;
- Parallel and split-mouth design studies including systemically healthy chronic periodontitis patients;
- The study reported on clinical treatment outcomes, including PD and/or CAL and/or BOP;
- If multiple antimicrobials were tested, outcomes were reported separately for each agent;
- English language.

At the second stage of selection, all full-text articles identified during the first stage were acquired and evaluated according to the following exclusion criteria:

- Studies including patients with systemic diseases;
- Studies where aggressive periodontitis patients were treated;
- Studies where antiseptics were continuously reapplied to progressing tooth sites or applied before the initial periodontal treatment;
- Studies where SRP was performed only with ultrasonic instruments;
- Studies not reporting on the clinical treatment outcomes, including changes in CAL and/or PD and/or BOP.

### Data extraction

Data extraction templates were used to retrieve general information on the country, study design, periodontal status of included cohorts, follow-up periods, number of patients, patients’ gender, age, smoking status, and tested products (Table 1). The number of patients at baseline and at end of the study, periodontal case definitions, treatment protocols, and clinical outcomes are presented in Table 2. The mean values and standard deviations of changes in PD and BOP reduction and in CAL gain following the treatment in test and control groups were extracted for the data analysis (Table 2). In cases where a study did not report exact data of interest but included precise graphic representations, data were extracted.

**Table 1:** Material and methods of the selected studies: country, study design, periodontal status of included cohort, follow-up, sample size, gender, smoking status, age and tested product

Study	Country	Study design	Periodontal status	Follow-up	Number, gender	Smokers	Mean (range) age	Product tested
Bizzarro S. et al., 2017 [13]	Holland	Parallel RCT	CP	12 months	56(36 M, 20F)	Included	47.8 ± 9.3	0,5% NaOCl solution
Kanoriya D. et al., 2017 [14]	India	Parallel RCT	CP	6 months	42(NR)	Excluded	22–55	0,75% boric acid gel
Denez E.M. et al., 2016 [14]	Belgium	Split-mouth RCT	Moderate-Severe CP	6 months	28(NR)	Excluded	45 ± 9.7	10% PVI solution
Matesanz P. et al., 2013 [20]	Spain	Parallel RCT	PD	6 months	22(8 M, 14F)	Included	50	CHX -xanthan gel
Krück C. et al., 2012 [21]	Germany	Parallel RCT	Moderate CP	12 months	51 (22 M, 29F)	NR	51 ± 11	0,12 CHX solution, 7,5% PVI solution
Sakellari D. et al., 2010 [15]	Greece	Parallel RCT	CP	6 months	56(25 M, 25 F)	Included	36–75	CHX chip
Paolantonio M. et al., 2009 [22]	Italy	Split-mouth RCT	Moderate – advanced CP	6 months	98(39 M, 59F)	Excluded	24–58	CHX-xanthan gel
Paolantonio M. et al., 2008 [23]	Italy	Split-mouth RCT	Moderate – advanced CP	6 months	82 (33 M, 49F)	Excluded	31–63	CHX chip
Paolantonio M. et al., 2008 [24]	Italy	Split-mouth RCT	C Moderate – advanced CP	6 months	116(34 M, 82F)	Excluded	33–65	CHX chip
Cosyn J. et al., 2007 [25]	Belgium	Parallel RCT	CP patients	6 months	33(16 M, 17 F)	NR	50.5 ± 12.5	CHX varnish
Azmak N. et al., 2002 [26]	Turkey	Split-mouth RCT	Moderate-Severe CP	6 months	22(NR)	Excluded	36–62	CHX chip
Heasman PA. et al., 2001 [27]	United Kingdom	Split-mouth RCT	Moderate-Severe CP	6 months	26 (8 M, 16 F)	Excluded	42.6 ± 12.6	CHX chip

*CHX* chlorhexidine gluconate

*CP* chronic periodontitis

*F* female

*M* male

*NaOCl* sodium hypochlorite

*NR* not reported

*PD* periodontal disease

*PVI* povidone iodine

**Table 2:** Material and methods of the selected studies: number of participants at baseline and end of the study, periodontal case definition, treatment protocols, changes in PD, CAL and BOP in test and control groups

Study	Participants	Periodontal case	Intervention	PD changes (mm) mean ± SD	CAL changes (mm) mean ± SD	BOP changes (%) mean ± SD	Comments
Bizzarro S. et al., 2017 [13]	CONTROL	≥2 non-adjacent teeth interproximal attachment loss of ≥3 mm;	1.SRP + S;	Control 1 ± 0.6;	Control 0.6 ± 0.5	Control 42.3 ± 16.9	**NS**
	Baseline *n* = 29;		2. SRP+ 0,5% NaOCl;	Test 0.9 ± 0.3	Test 0.5 ± 0.5	Test 41 ± 12.6	
	End of the study *n* = 29;	2 teeth per quadrant with PD ≥ 5 mm,					
	TEST	> 50% BOP;					
	Baseline *n* = 27;						
	End of the study *n* = 27.						
Kanoriya D. et al., 2017 [14]	CONTROL	PD ≥ 5 mm or CAL ≥4 mm and vertical bone loss ≥3 mm	Control: SRP + placebo gel;	Control 1.89 ± 0.45	Control 1,31 ± 0,82	–	Test group showed significant improvements in CAL gain and PD reduction
	Baseline *n* = 21;		Test: SRP + 0,75% boric acid gel.	Test 3.15 ± 0.74	Test 2.65 ± 0.58		
	End of the study *n* = 19;						
	TEST						
	Baseline *n* = 21;						
	End of the study *n* = 20.						
Denez E.M. et al., 2016 [26]	CONTROL	At least one pocket in each quadrant with PD ≥ 4 mm and BOP(+)	Control: SRP + 0,9% NaCl;	Control 1.92 ± 0.12	Control 1.93 ± 0.05	–	No significant difference between NaCl and 10%PVI in terms of clinical changes
	Baseline *n* = 28;		Test: SRP+ 10% PVI	Test 1.9 ± 0.3	Test 1.95 ± 0.21		
	End of study *n* = 20;						
	TEST						
	Baseline *n* = 28;						
	End of study *n* = 20.						
Matesanz P. et al., 2013 [20]	CONTROL	4–10 pockets with PD > 4 mm and BOP(+)	Control: SRP + placebo gel;	Control 0.22 ± 0,46	Control −0.01 ± 2,1	Control 15 ± 0.4	No significant difference between placebo and CHX xanthan gel in terms of clinical changes
	Baseline *n* = 12;		Test: SRP+ CHX xanthan gel	Test 0.32 ± 0.47	Test 0.3 ± 0.98	Test 18 ± 0.4	
	End of study *n* = 11;						
	TEST						
	Baseline *n* = 10;						
	End of study *n* = 10.						
Krück C. et al., 2012 [28]	CONTROL	PD 4-6 mm.	Control: SRP + 0,9% NaCl;	Control 0.36 ± 0.4	Control 0.21 ± 0.7	Control 16 ± 15	No significant difference between NaCl, 0,12% CHX and 7,5 PVI in terms of clinical changes
	Baseline *n* = 17;		Test 1: SRP+ 0,12% CHX;	Test 1: 0.38 ± 0.4	Test1: 0.22 ± 0.65	Test 1: 18 ± 17	
	End of study *n* = 17;		Test 2: SRP + 7,5% PVI	Test 2: 1.39 ± 0.42	Test2: 0.36 ± 0.5	Test 2: 25 ± 17	
	TEST1						
	Baseline *n* = 17;						
	End of study *n* = 17;						
	TEST2						
	Baseline *n* = 17;						
	End of study *n* = 17.						
Sakellari D. et al., 2010 [15]	CONTROL	PD ≥5 mm, ≤7 mm.	Control: SRP;	Control 2.05 ± 0.74	Control 1.4 ± 0.71	Control 33 ± 32	NS between test and control groups
	Baseline *n* = 29;		Test: SRP+ CHX chip.	Test: 1.79 ± 0.84	Test: 1.4 ± 0.97	Test: 25 ± 33	
	End of study *n* = 25;						
	TEST						
	Baseline *n* = 27;						
	End of study *n* = 25.						
Paolantonio M. et al., 2009 [22]	CONTROL	At least 2 teeth with PD ≥ 5 mm	Control: SRP;	Control 1.5 ± 0.15	Control 0.51 ± 0.11	–	Significantly greater PD and CAL improvements in test group
	Baseline *n* = 98;		Test: SRP+ CHX-xanthan gel	Test: 2.33 ± 0.15	Test: 1.41 ± 0.11		
	End of study *n* = 98;						
	TEST						
	Baseline *n* = 98;						
	End of study *n* = 98.						
Paolantonio M. et al., 2008 [24]	CONTROL	2 or more teeth with PD PD ≥ 5 mm, and BOP(+)	Control: SRP;	Control 1.9 ± 1.95	Control 0.9 ± 1.9	–	Significantly greater PD reduction and CAL gain in test group
	Baseline *n* = 82;		Test: SRP + CHX chip	Test: 2.7 ± 1.44	Test: 1.4 ± 1.2		
	End of study *n* = 82;						
	TEST						
	Baseline *n* = 82;						
	End of study *n* = 82.						
Paolantonio M. et al., 2008 [23]	CONTROL	At least 2 teeth with PD ≥ 5 mm	Control: SRP;	Control 0.95 ± 0.1	Control 0.49 ± 0.1	–	Significantly greater PD and CAL improvements in test group
	Baseline *n* = 116;		Test: SRP + CHX chip	Test: 1.5 ± 0.1	Test: 1.13 ± 0.1		
	End of study *n* = 116;						
	TEST						
	Baseline *n* = 116;						
	End of study *n* = 116.						
Cosyn J. et al., 2007 [25]	CONTROL	At least 1 pocket per quadrant with PD ≥ 6 mm, BOP(+), radiographic evidence of extended bone loss (≥1/3 of the root length	Control: SRP;	Control 0.96 ± 0.43	Control 0.39 ± 0.78	Control 30 ± 15	NS
	Baseline *n* = 16;		Test: SRP+ CHX varnish.	Test: 1.13 ± 0.62	Test: 0.36 ± 0.93	Test: 34 ± 20	
	End of study *n* = 14;						
	TEST						
	Baseline *n* = 17;						
	End of study *n* = 15.						
Azmak N. et al., 2002 [27]	CONTROL	At least 2 non-adjacent interproximal sites in the anterior region with PD 6–8 mm, BOP(+);	Control: SRP	Control 2.1 ± 0.2	Control 1.56 ± 0.21	–	NS
	Baseline *n* = 22;		Test: SRP+ CHX chip	Test: 2.4 ± 0.2	Test: 1.68 ± 0.21		
	End of study *n* = 20;						
	TEST						
	Baseline *n* = 22;						
	End of study *n* = 20.						
Heasman PA. et al., 2001 [21]	CONTROL	At least one pocket per quadrant with PD ≥5 mm, BOP(+)	Control: SRP	Control 0,45 ± 0,13	Control 0.15 ± 0.09	Control 45 ± 13	Significantly greater improvements in all clinical parameters in test group
	Baseline *n* = 26;		Test: SRP+ Perio chip	Test 0,78 ± 0,12	Test: 0.43 ± 0.15	Test: 78 ± 12	
	End of study *n* = 24;						
	TEST						
	Baseline *n* = 26;						
	End of study n2 = 24.						

*BOP* bleeding on probing

*CAL* clinical attachment level

*CHX* chlorhexidine gluconate

*NaOCl* sodium hypochlorite

*NS* no significant difference between test and control groups

*PD* probing depth

*PVI* povidone iodine

*SRP* scaling and root planing

When the differences (∆) between baseline-end visits were not reported, they were calculated according to the formula: ∆Vary = Var2-Var1 (Var1 and Var2 – mean values before and after treatment). The variance was estimated with the formula: SVar^2^ = SVar1^2^- SVar2^2^ – (2*r*SVar1*SVar2), (SVar1^2^ and SVar2^2^ – variances of the mean baseline and end values) [29]. A correlation r of 0,5 was assumed [30].

### Risk of bias assessment

The quality of all included studies was assessed during the data extraction process and involved an evaluation of the methodological elements that could influence each study’s outcome (Table 3). The Cochrane Collaboration’s 2-part tool for assessing the risk of bias [31] was used to assess bias across the studies and to identify papers with intrinsic methodological and design flaws. The following items were evaluated as posing a low, high, or unclear risk of bias: 1) random sequence generation, 2) allocations concealment, 3) the blinding of participants/personnel, 4) incomplete outcome data, 5) selective reporting outcomes, 6) other potential risks of bias. The degree of bias was categorized as low risk if all criteria were met, moderate risk when one criterion was missing, and high risk if two or more criteria were missing.

**Table 3:** Assesment of the risk of bias

Author, year	Random sequence generation	Allocation concealment	Blinding	Incomplete outcome data	Selective reporting	Other bias
Kanoriya D. et al., 2017 [14]	+	?	+	+	+	+
Bizzarro S. et al., 2017 [13]	+	+	+	+	+	+
Denez E.M. et al., 2016 [14]	+	–	–	+	+	+
Matesanz P. et al., 2013 [20]	+	+	+	+	+	+
Krück C. et al., 2012 [21]	–	+	–	+	+	+
Sakellari D. et al., 2010 [15]	+	+	+	+	+	+
Paolantonio M. et al., 2009 [22]	+	–	+	+	+	+
Paolantonio M. et al., 2008 [23]	+	–	+	+	+	+
Paolantonio M. et al., 2008 [24]	+	–	+	+	+	+
Cosyn J. et al., 2007 [25]	+	–	+	+	+	+
Azmak N. et al., 2002 [26]	+	–	–	+	+	+
Heasman PA. et al., 2001 [27]	–	–	–	+	+	+

+ = Low risk? = Unclear risk - = High risk

### Statistical analysis

All meta-analyses were performed on randomized controlled clinical trials that reported the clinical outcomes of nonsurgical periodontitis treatment utilizing various adjunctive antiseptics.

Individual trials were pooled, and the overall rates of probing-depth reduction, clinical attachment level gains, bleeding-on-probing reduction, and the 95% confidence intervals (CIs) among the treatment groups were calculated. Fixed or random effects models were used based on the presence or absence of heterogeneity among the included studies. The heterogeneity among the included trials was tested by the heterogeneity test using the Cochran Q statistics. In our case the random-effects model (the Der Simonian and Liard method) [32] was more eligible, as it tended to give a more more conservative estimate, nevertheless the results of both models usually agreed well. An unweighted kappa index was used to evaluate the level of agreement between 2 independent researchers.

## Results

### Study selection

**Fig. 1:**
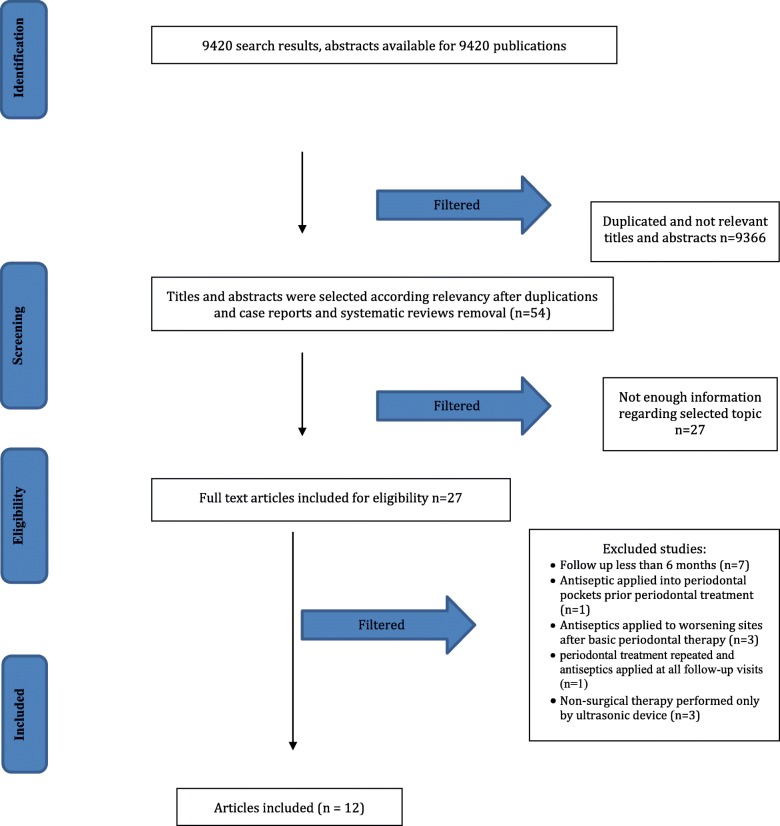
PRISMA flow diagram

The initial search resulted in 9420 articles from the MEDLINE (Ovid) and Cohrane Central Register of Controlled Trials (CENTRAL) databases. After evaluating titles and abstracts, inclusion and exclusion criteria were applied to the remaining 27 full-text articles (inter-reader agreement κ = 0.85). Finally, 12 RCTs were included into the review (inter-reader agreement κ = 0.96). The study selection process is presented in Fig. 1.

### Study exclusion

The reasons for excluding studies after full-text assessment were as follows: a follow-up time < 6 months (*n* = 7) [33–39], antiseptics applied to periodontal pockets prior to periodontal treatment (*n* = 1) [40], antiseptics applied continuously to worsening sites after an initial periodontal treatment (*n* = 3) [41–43], repeated periodontal treatment and antiseptics applied at all follow-up visits (*n* = 1) [44], and periodontal treatment established only by ultrasonic instruments (*n* = 3) [45–47].

### Quality assessment

To summarize the risk of bias for each study, 3 studies were classified as having a low risk of bias (all domains included) [13, 15, 20], 5 studies had a moderate risk (bias for 1 key domain [14, 22–25], and 4 studies were judged to have a high risk of bias (more than 1 domain) [14, 21, 26, 27].

### Study design

The included studies are described in Table 1. Six studies used a split-mouth design [14, 22–24, 26, 27], whereas the remaining investigations had a parallel arms design [13–15, 20, 21, 25]. The follow-up period ranged from 6 (10 studies [14, 15, 20, 22–27]) to 12 months (2 studies [13, 21]). Two of the studies had more than one test group (i.e., two [21] and three [13] test groups). However, due to the adjunctive use of systemic antibiotics following the SRP, only one test group of one of the aforementioned studies [13] was included in the current analysis.

### Study population

The present analysis involved a total of 632 consecutive periodontal patients [13–15, 21–26] and patients enrolled in a regular periodontal maintenance program [20, 27]. In total, 606 (95.8%) patients completed the studies. The mean age of the included patients ranged from 22 [14] to 75 years [15], and the ratio of included males and females varied from 0.30 [23] to 0.67 [27]. Ten studies [13–15, 20–25, 27] were based on patient samples from a European population, and 2 studies [14, 26] were based on an Asian population.

Two studies [21, 25] did not report on patient smoking habits, and smokers were excluded in 7 studies [14, 22–24, 26, 27]. In 3 investigations [13, 15, 20] that included smoking patients, the proportion of smokers ranged from 16% [15] to 55% [13].

Patient-related data are depicted in Table 1.

### Antiseptics

Table 1 shows antiseptic materials adjunctively applied during the SRP. Studies were divided into 2 broad groups based on the vehicle employed to deliver the antiseptic agent: adjunctive sustained-release antiseptics (gels, chips, and varnish [14, 15, 20, 22–27]) and adjunctive irrigation with antiseptics (antiseptic delivered by syringe [13, 14, 21]).

### Interventions

Treatment protocols used in the test and control groups are depicted in Table 2. Full-mouth SRP was accomplished in all studies before the application of tested materials. Two studies [14, 20] used placebos in the control groups. In all studies, oral hygiene instructions were given to the patients prior to treatment, and oral hygiene was reinforced at each follow-up visit. Except for 1 study [13] where patients were prescribed to rinse with 0.12% chlorhexidine, additional postoperative antiseptic rinsing was restricted in the remaining studies.

### Synthesis of results

Meta-analyses were performed only if studies with similar comparisons reported the same outcome measures.

In spite of a high heterogeneity among the included studies (I^2^ = 97%, *p* = 0.001), an evaluation of the overall effect of antiseptics used as adjuncts to SRP showed statistically significant changes for the PD (*p* = 0.001; SMD = 1.536, 95% CI = 0.402 to 2.670), CAL (*p* = 0.001; SMD = 1.515, 95% CI = 0.289 to 2.741), and BOP (*p* = 0.001; OR = 0.995, 95% CI = 0.0761 to 1.913) values irrespective of the antiseptics delivery vehicle compared to SRP alone.

**Fig. 2:**
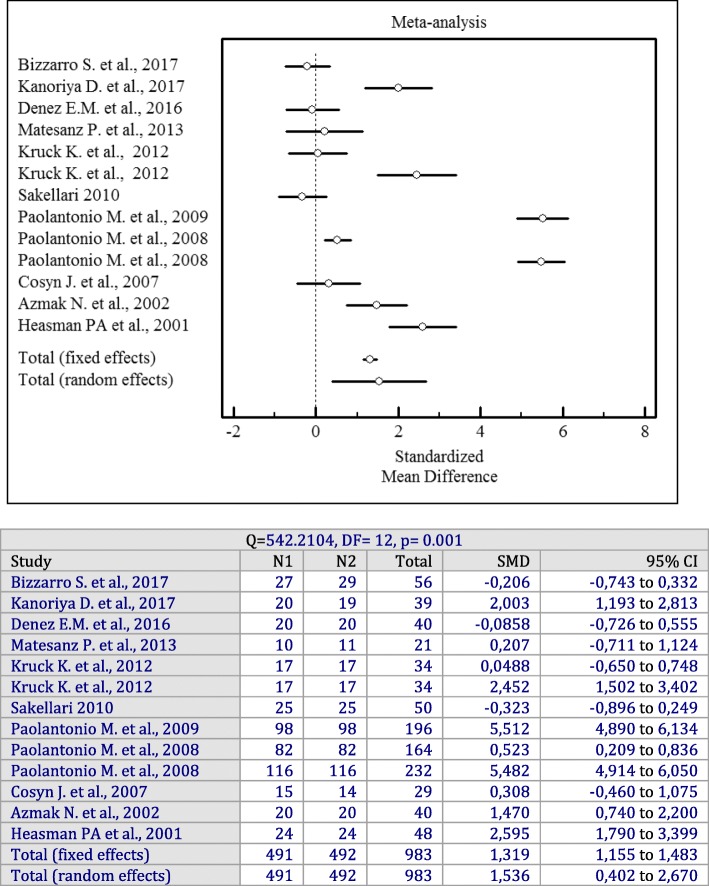
Forest plot of odds ratio (95% CI) for probing depth using adjunctive antiseptics

**Fig. 3:**
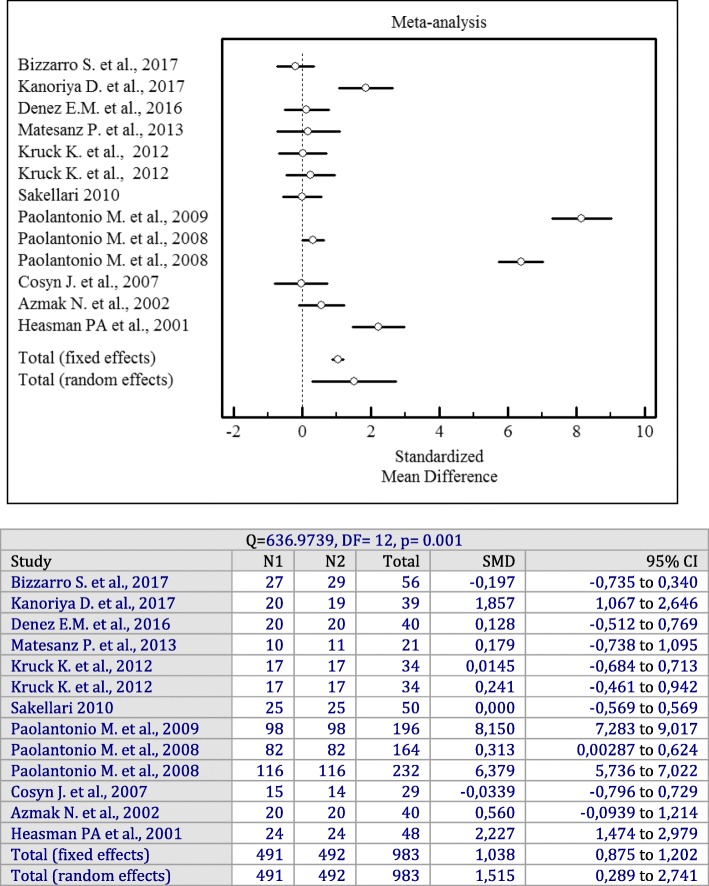
Forest plot of odds ratio (95% CI) for clinical attachment level using adjunctive antiseptics

**Fig. 4:**
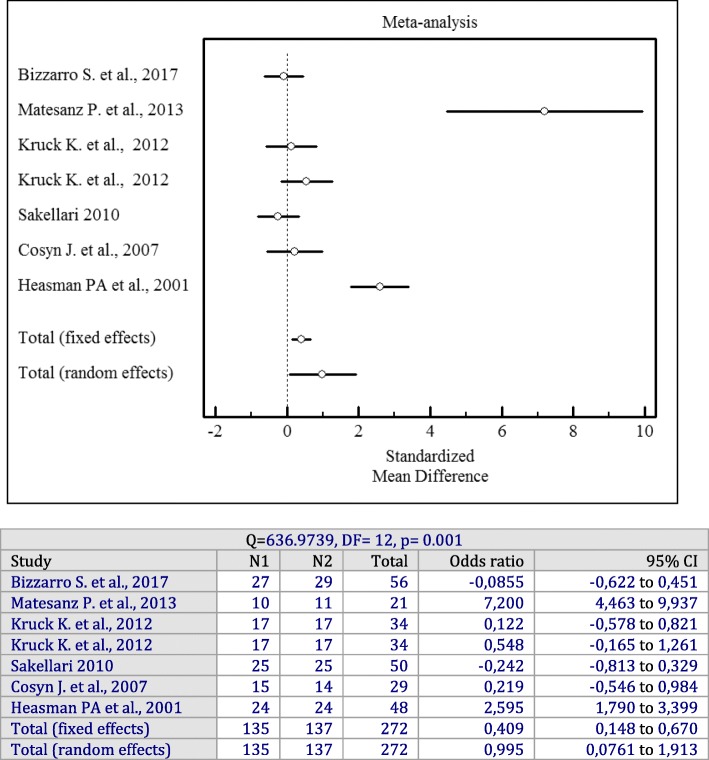
Forest plot of odds ratio (95% CI) for bleeding on probing using adjunctive antiseptics

Forest plots of odds ratios (95% CI) for PD, CAL, and BOP using adjunctive antiseptics with SRP are demonstrated in Figs. 2, 3 and 4.

### Adjunctive sustained-release antiseptics

Nine studies [14, 15, 20, 22–27] with 405 patients were included in a meta-analysis for PD and CAL changes. The meta-analysis found that a sustained-release delivery of antiseptics resulted in a significantly greater PD reduction compared to SRP alone (*p* = 0.001). There was significant heterogeneity among studies (I^2^ = 98%, Q = 454.9179, df = 8, *p* = 0.001, SMD = 1.977 mm; 95% CI: 0.470 to 3.485).

Likewise, when considering the CAL changes, a sustained-release delivery system of antiseptics demonstrated statistically significant greater gains in CAL compared to SRP alone (*p* = 0.001). There was significant heterogeneity among studies (I^2^ = 98%, Q = 576.4, df = 8, *p* = 0.001, SMD = 2.174 mm; 95% CI: 0.438 to 3.909).

Four studies with 124 patients were included in a meta-analysis for the changes of BOP [15, 20, 25, 27]. Its findings pointed to a statistically significant higher reduction in BOP scores when sustained-release antiseptics were applied compared to SRP alone (*p* = 0.001). Significant heterogeneity among the studies was found (I^2^ = 94%, Q = 59.8429, df = 3, *p* = 0.001, OR = 2.028; 95% CI: 0.119 to 3.936).

**Fig. 5:**
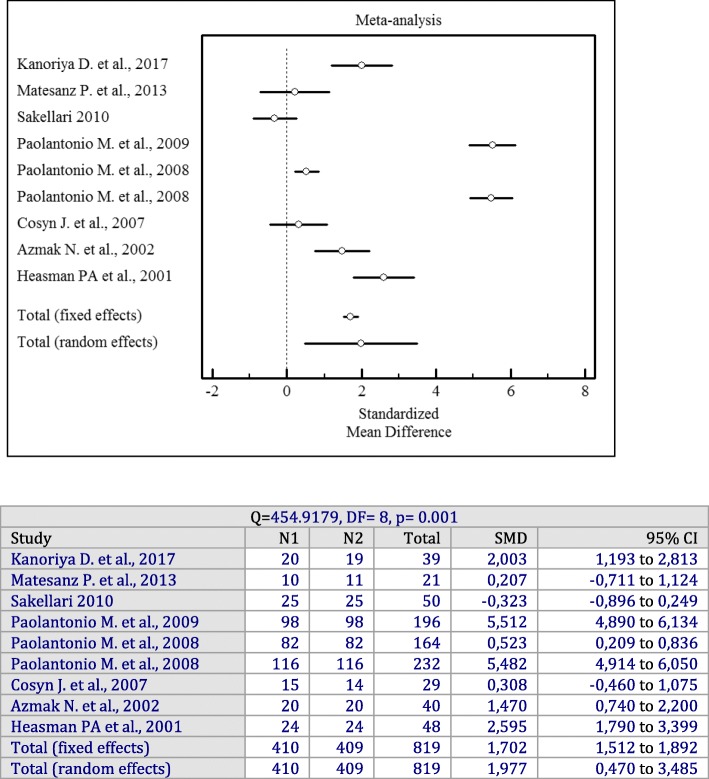
Forest plot of odds ratio (95% CI) probing depth reduction using adjunctive sustained-release vehicle antiseptics

**Fig. 6:**
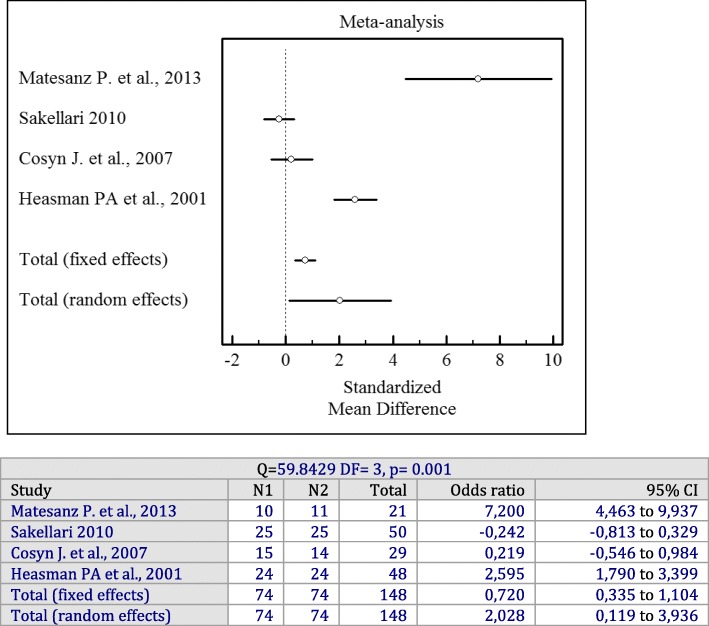
Forest plot of odds ratio (95% CI) for bleeding on probing reduction using adjunctive sustained-release vehicle antiseptics

**Fig. 7:**
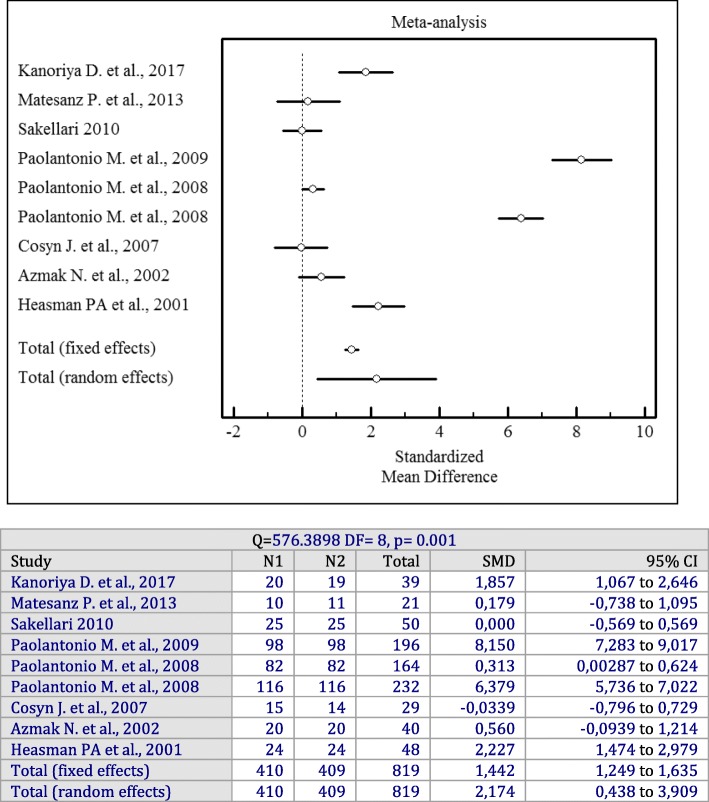
Forest plot of odds ratio (95% CI) for clinical attachment gain using adjunctive sustained-release vehicle antiseptics

Forest plots of odds ratios (95% CI) for PD, BOP reduction, and CAL gains using adjunctive antiseptics for scaling and root planning in a sustained-release vehicle are demonstrated in Figs. 5, 6 and 7.

### Adjunctive irrigation with antiseptics

For the meta-analysis evaluating the effectiveness of adjunctive subgingival irrigation with antiseptics in terms of PD and CAL changes, 3 studies with 127 patients were included [13, 14, 21]. The results demonstrated that a liquid form of subgingivally applied antiseptics did not significantly change PD values compared to SRP alone (*p* = 0.321). There was significant heterogeneity among studies (I^2^ = 89%, Q = 27.3343 df = 3, *p* = 0.321, SMD = 0.460 mm; 95% CI: − 0.546 to 1.467).

Similarly, liquid forms of antiseptics did not significantly change CAL compared to SRP alone (*p* = 0.7568). There was no significant heterogeneity among studies (I^2^ = 0%, Q = 1.1843, df = 3, *p* = 0.7568, SMD = 0.0169; 95% CI: − 0.292 to 0.326).

Based on the two studies with 107 patients, the additional application of a liquid form of antiseptics did not significantly reduce BOP as compared to SRP alone (*p* = 0.3549) [13, 21]. These studies did not demonstrate significant heterogeneity (I^2^ = 7%, Q = 2.07, df = 2, *p* = 0.3549, OR = 0.141; 95% CI: − 0.217 to 0.499).

**Fig. 8:**
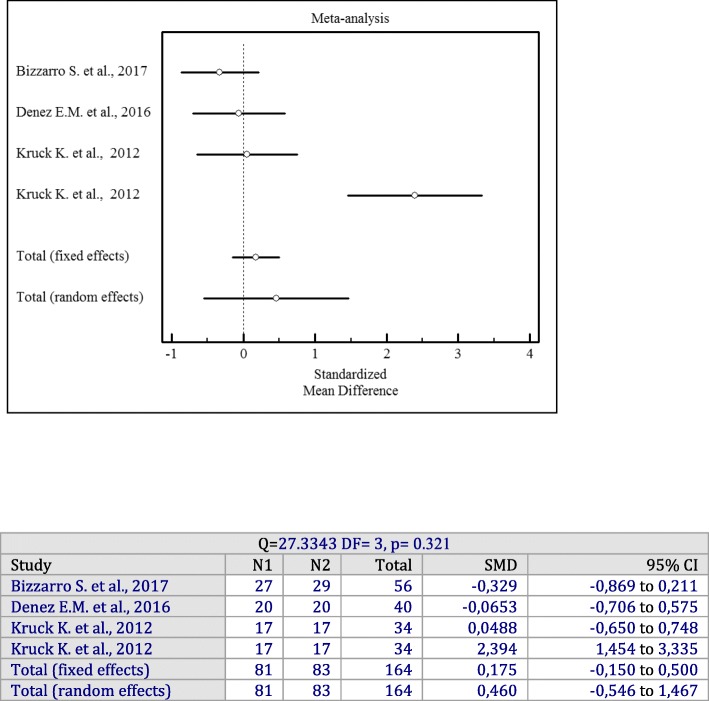
Forest plot of odds ratio (95% CI) for probing depth reduction using adjunctive irrigant antiseptics

**Fig. 9:**
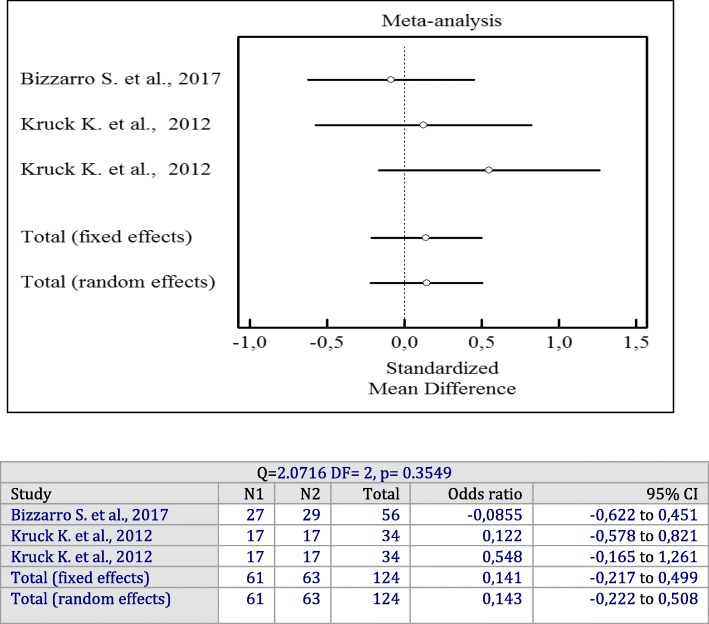
Forest plot of odds ratio (95% CI) for bleeding on probing using adjunctive irrigant antiseptics

**Fig. 10:**
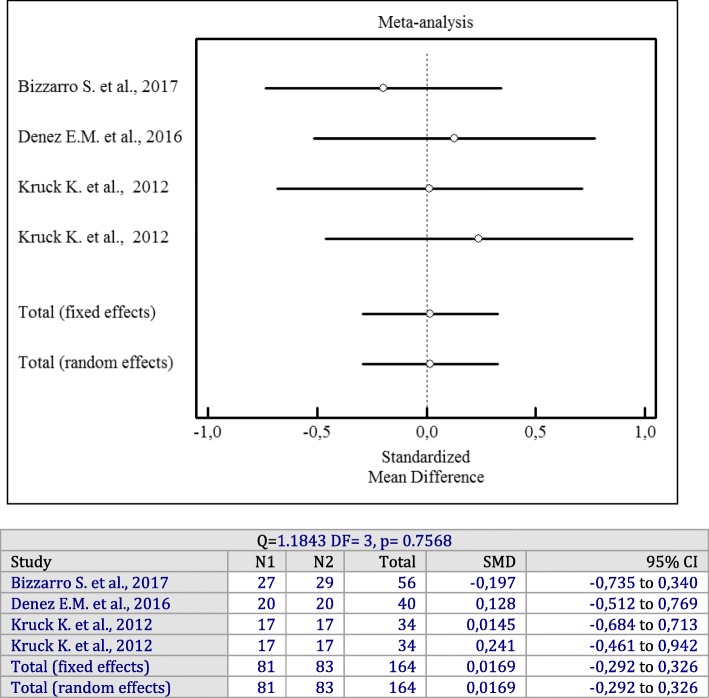
Forest plot of odds ratio (95% CI) for clinical attachment level gain using adjunctive irrigant antiseptics

Forest plots of odds ratios (95% CI) for PD, BOP reduction, and CAL gains using adjunctive antiseptics for scaling and root planning in additional irrigation studies are demonstrated in Figs. 8, 9 and 10.

## Discussion

The present study aimed to investigate the potential beneficial effects of the adjunctive application of subgingivally delivered antiseptics to SRP for treating periodontitis patients.

Based on our findings, the overall use of adjuvant antiseptics brings an additional clinical advantage compared to SRP alone. The meta-analysis demonstrated significant improvements in PD, CAL values, and BOP scores following the subgingival application of antiseptics compared to the control (SRP alone) (*p* = 0.001). However, these improvements were shown to depend on the antiseptics’ delivery vehicle. Particularly, only antiseptics with a sustained-release vehicle (gels, chips, and varnish) were found to have significant clinical improvements in terms of PD, BOP reduction, and CAL gain (*p* = 0.001). The addition of subgingival irrigation with antiseptics failed to show significant improvement of clinical parameters compared to the controls (*p* > 0.05).

The effectiveness of using adjunctive local antiseptics in combination with SRP was evaluated in previous systematic reviews [29, 48–50]. Accordingly to our findings, different CHX concentrations using various administration vehicles (CHX chips, CHX varnish, and CHX plus xanthan gel) showed an overall significant effect with significant differences (*p* = 0.000) for changes in PD and in CAL compared to SRP alone [29, 48]. Moreover, a positive adjunctive effect (i.e., significant PD reduction (*p* = 0.058) and CAL gains (*p* = 0.015)) of sustained-release antiseptics (CHX chips), but not irrigated (CHX solution), in combination with SRP was demonstrated [50].

The aforementioned systematic reviews also evaluated adjunctive benefits of locally delivered antibiotics with a sustained-release delivery [29, 48–50]. Their clinical efficacy in terms of PD and BOP reduction and CAL gain was comparable to the efficacy obtained with sustained-release antiseptics. However, it was previously highlighted that the use of locally delivered antibiotics should be limited [16]. Therefore, we did not intend to investigate the clinical benefits of adjuvant local antibiotics with SRP in the current review.

Two of the included studies found the initial PD values to be associated with the treatment outcomes [14, 25]. In particular, initial probing depths of 6 mm and 7 mm were shown to result in significantly greater PD reductions when compared with the baseline PD values of < 5 mm [14, 25]. As stated in a study by Salvi et al. [51], a PD reduction of 2 mm to 2.5 mm in sites exceeding 6 mm at baseline is to be expected. Therefore, additional pocket reduction would represent a true clinical benefit of adjunctive therapies [51]. Aforementioned studies [14, 25] found extra PD reduction in initially deep sites in favor of test groups (0.93 and 2 mm respectively), thus indicating a clinical advantage of adjunctive antiseptics in deep periodontal pockets.

A current investigation of various antiseptic materials analyzed their various formulations and delivery forms, which did not allow us to subgroup the studies according to antiseptic agents. This suggests the need for well-designed, long-term randomized controlled clinical trials utilizing antiseptics as adjuncts to SRP in the treatment of periodontitis.

This systematic review was limited to only randomized controlled clinical studies. The current review only included studies written in English, which could introduce a publication bias.

Only 3 [13, 15, 20] out of 12 studies had a low risk of bias, which included relatively small number of patients. Other studies were evaluated as having a moderate (*n* = 5) or high (*n* = 4) risk of bias. These aspects are important for detecting methodological weaknesses of the included studies that might alter therapy outcomes. According to the results of a bias risk assessment, allocation concealment and the blinding of participants and personnel appeared to be the most critical domains.

In the current review, data for investigated primary and secondary outcome variables showed a high degree of heterogeneity (> 85%). Factors that impact it might include differences of the studied populations, differences in disease severity, the therapeutic agent’s type and concentration, and the location of defects, which makes it difficult to evaluate the real effect of tested products.

Only studies with a follow-up of no less than 6 months were included in the review; thus, results from studies with a shorter follow-up period were not included in the analysis, which might influence our results. For example, Matesanz et al. [20] found statistically significant PD reductions in short-term studies (studies with a follow-up of less than 6 months) for additional CHX chips (*n* = 7) and CHX varnish (*n* = 2) and significant CAL gains for CHX chips (*n* = 8).

Analyzed studies included relatively small number of patients, thus questioning if the real effect of tested materials could be detected. In particular, 3 studies [22–24] with big study cohorts found significant clinical improvements for the adjunctive use of subgingival antiseptics, suggesting the need for large trials encompassing bigger study cohorts.

## Conclusions

Based on the findings of the current systematic review, adjunctive subgingivally delivered antiseptics with a sustained-release delivery have significant clinical benefits compared to SRP alone. Furthermore, future studies should be based on adequate methodological procedures to improve the overall quality of the reporting and to reduce the risk of bias.

## Data Availability

All data generated or analysed during this study are included in this published Article.

## Abbreviations

BOP: Bleeding on probing
CAL: Clinical attachment level
CHX: Chlorhexidine digluconate
CI: Confidence intervals
DF: Degrees of freedom
PD: Probing depth
SMD: Standard mean deviation
SRP: Scaling and root planing
